# Predicting breakthrough of vanadium in fixed-bed absorbent columns with complex groundwater chemistries: A multi-component granular ferric hydroxide−vanadate−arsenate−phosphate−silicic acid system

**DOI:** 10.1016/j.wroa.2020.100061

**Published:** 2020-08-05

**Authors:** Anna Dabizha, Carsten Bahr, Michael Kersten

**Affiliations:** a Geosciences Institute, Johannes Gutenberg-University, Becherweg 21, Mainz, 55099, Germany; b GEH Wasserchemie GmbH & Co. KG, Adolf-Köhne-Straße 4, Osnabrück, 49090, Germany

**Keywords:** Vanadate, Adsorption, FBA, HSDM, RSCCT, CD-MUSIC

## Abstract

Granular ferric hydroxide (GFH) is often used for fixed bed adsorbent (FBA) columns in groundwater purification units around the world to remove arsenate contaminations. Groundwater can contain also other toxic (e.g., antimonite and vanadate) and non-toxic oxo-anions (phosphate and silicic acid) that are known to affect FBA lifetimes. Therefore, understanding the breakthrough of toxic compounds intended for removal by FBA is essential to their design, and is important to predict accurately breakthrough curves (BTCs) for FBAs in waterworks to plan future operating costs. Rapid small-scale column tests (RSCCT) and pilot-scale FBA were used to simulate vanadate BTCs for complex groundwater chemistries. The BTCs were simulated successfully using a homogeneous surface diffusion model (HSDM) combining equilibrium chemical adsorption and kinetic mass transfer. Adsorption parameters for various groundwater compositions were predicted using the CD-MUSIC surface complexation model, which was set up for the first time for akaganéite-based granular ferric hydroxide with a competitive multi-solute system. The results indicated that V(V) is less prone to competitive adsorption effects, and use of the homogeneous surface diffusion model to predict the BTCs requires then the kinetic mass transfer Biot number to be used as the only fitting parameter. On the other hand, a concentration overshoot could be observed for the two weaker absorbed oxo-anions arsenate and phosphate because of displacement by the vanadate. Results of pilot scale test column BTCs of vanadate for three waterworks with different groundwater compositions could be favorably extrapolated with a unique Freundlich constant k_F_ of 3.2 derived on basis of the multi-solute CD-MUSIC model, and a unique Biot number of 37 fixed for all three different test sites.

## Highlights

• Rapid small-scale and pilot-scale column tests were used to simulate vanadate BTCs.
• Freundlich constants derived on basis of a multi-solute CD-MUSIC model.
• BTC modelling required the Biot number to be used as the only fitting parameter.
• Vanadate is less prone to competitive multi-solute adsorption effects.
• Same Freundlich and Biot numbers could be derived for three different test sites.

## Graphical abstract

**Figure:**
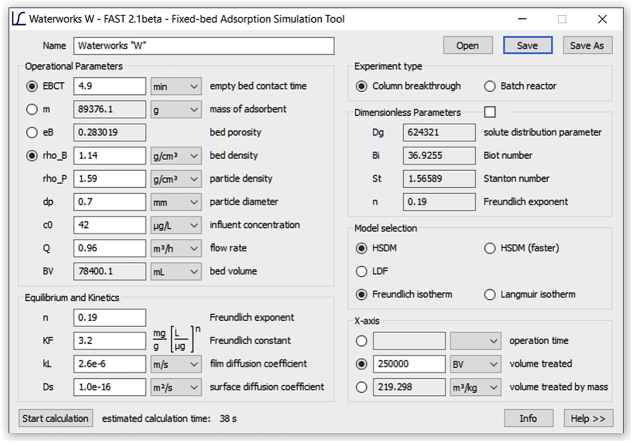

## 1 Introduction

Ferric hydroxide-based adsorbents can be used very efficiently to remove hazardous oxo-anions from contaminated groundwater (Weidner and Ciesielczyk, 2019). Such materials are often used as filler in fixed-bed adsorbent (FBA) columns for water purification facilities (Deliyanni et al., 2003, 2009; Daus et al., 2004; Badruzzaman et al., 2004; Saha et al., 2005; Sperlich et al., 2005, 2008; Naeem et al., 2007; Banerjee et al., 2008; Streat et al., 2008; Guan et al., 2008; Kolbe et al., 2011; Hilbrandt et al., 2019). FBAs packed with granular ferric hydroxide (GFH) have high adsorption capacities, but when used for a long time the adsorbent may become saturated or even overloaded and the contaminant concentrations in the effluent increase above acceptable values. The adsorbent used in an FBA must therefore be replaced regularly to keep contaminant concentrations below the relevant thresholds (e.g., in Germany 10 μg L^−1^ for As and 4 μg L^−1^ for V: LAWA, 2004). This implies limited lifetimes of typically 100–1000 d, whereby 20,000–300,000 bed volumes (BVs) of groundwater can typically be treated (Worch, 2012). In designing an FBA, breakthrough for the toxic compounds to be removed must be properly understood for a vast variety of groundwater compositions, which may enable for accurate predictions of breakthrough curves (BTCs) as important prerequisite to allow waterworks staff to estimate future operating costs.

Unfortunately, it is not just trivial to predict the lifetime of an FBA with any confidence because of the complex interaction of the many co-factors and competitive co-adsorbates in groundwater (Pincus et al., 2020). For example, a small change in pH can markedly affect the mobility of oxo-anions such as As(III) and As(V) in contaminated groundwater (Biswas et al., 2014). As many oxo-anions share similar adsorbate structures, their adsorptive mechanisms also tend to be alike, thus promoting competition for adsorption sites. Background oxo-anions such as phosphate (PO_4_^−^^3^) and silicic acid (H_4_SiO_4_^0^) are known to compete with the trace As for similar adsorption sites, contributing to failure in achieving regulatory limits for the target contaminant within expected lifetime of an FBA (Nguyen et al., 2011). While there is lots of information about adsorption selectivity and competition between the oxo-anions arsenite/arsenate, selenite/selenate, phosphate, and silicic acid (Pincus et al., 2020), there has been less focus on selective and competitive adsorption behavior of the target contaminant vanadate. Elevated vanadium concentrations of up to 0.2 mg L^−1^ were found in groundwater of volcanic areas throughout the world (Hamada, 1998; Arena et al., 2015; Koh et al., 2016). V-bearing fluorapatite with up to 5 wt.-% of the phosphate substituted by vanadate was recently found as geogenic source (Härter et al., 2020). This implies also high phosphate concentrations, but their effect on vanadate removal by FBAs is virtually unknown. Enhanced V concentrations may also correlate with enhanced silicic acid and As concentrations in Tertiary and Quaternary fluvial and aeolian (alluvial) deposits in Argentina, where the potential sources were found to be layers of volcanic ash with 90% of rhyolitic glass dispersed in the sediments (Bundschuh et al., 2004; Battacharya et al., 2006). Individual and combined effects on adsorbents such as GFH under environmentally relevant conditions have been studied systematically in only a few laboratory studies (Nguyen et al., 2011; Kanematsu et al., 2012; Hilbrandt et al., 2019), and only in one of them vanadate was considered outcompeting the As adsorption by GFH (Kolbe et al., 2011).

However, not only a multitude of chemical groundwater components may affect the lifetime of FBAs, but also physical parameters like the empty bed contact time (EBCT). The EBCT is defined as the BV divided by the volumetric flow rate. The arsenate removal rate for an FBA has been found to be improved by decreasing the flow rate (Vaughan et al., 2007; Zeng et al., 2008). The lifetime in terms of the number of BVs (defined as the volume of water treated divided by the adsorbent bed volume) may reach tens to hundreds of thousands before the adsorbent needs to be replaced, meaning that an FBA may be used continually for two or more years. High adsorption capacities require large volumes of water or extremely high initial contaminant concentrations in feasibility studies using pilot-scale test columns in waterworks. It is therefore laborious and time-consuming to conduct field-scale experiments to study competitive ion interactions for lifetime estimation. However, rapid small-scale column tests (RSSCTs) have been used successfully to simulate the BTCs for full-scale FBA columns (Crittenden et al., 1987; Westerhoff et al., 2005; Sperlich et al., 2005, 2008; Nguyen et al., 2011; Kanematsu et al., 2012).

Systematic investigations of the effects of different pH values and phosphate, silicic acid, and vanadate concentrations on As breakthrough are scarce (Nguyen et al., 2011; Kolbe et al., 2011; Kanematsu et al., 2012). The pH and oxo-anion concentrations used in the study first cited were selected to reflect environmental conditions. Three commercially available adsorbents (including a goethite-based GFH and an akaganéite-based GFH) used to remove As were tested. A factorial experimental design was used in which all oxo-anion concentrations and pH were varied so that a relatively small number of column runs were required. Nonetheless, the number of runs was still challenging, and a major finding was that the akaganéite-based GFH was more susceptible than the goethite-based GFH to changes in water quality under the conditions tested. Although the column efficiency in terms of adsorption capacity was better for the akaganéite-based GFH, it was affected most strongly by pH due to the by one unit lower pH point-of-zero-charge (pH_PZC_ = 9.2 for goethite-based GFH vs. 8.2 for akaganéite-based GFH). A decrease in pH of around a half unit (from 7.6 to 7.0) caused a doubling of the number of BVs required for 10 μg L^−1^ arsenic breakthrough (As-BV_10_) for the akaganéite-based GFH (and vice versa), but an insignificant effect on goethite-based GFH. It is not surprising that a low pH favored oxo-anion removal, but it is intriguing to note the greater sensitivity of As-BV_10_ to changes in the influent As concentration at the lower pH for both GFH adsorbents. In fact, the As-BV_10_ was more affected by a change in pH when the inflow As concentration was 15 μg L^−1^ than when it was 35 or 55 μg L^−1^ (Nguyen et al., 2011). The main conclusion of this study was that water quality affects adsorbent media in different and hardly predictable ways, and column tests must therefore be performed using local sources of water before selecting an appropriate adsorbent, and before the operational and maintenance costs can be estimated (Nguyen et al., 2011). Nonetheless, this approach is severely hampered by the fact that laboratory-scale column test results cannot as easily be extrapolated to conditions other than those of the original experiment.

An alternative approach to quantify target contaminant selectivity is equilibrium surface complexation modelling (SCM). Not only antagonistic but also synergistic effects can occur. For example, Ca^2+^ mitigates the effects of increasing pH by decreasing the total surface negative charge (Stachowicz et al., 2008). Such charge effects can only be simulated using an advanced SCM approach such as the charge distribution (CD) multi-site surface complexation (MUSIC) model (Stachowicz et al., 2008). Unlike the adsorption isotherms used by Nguyen et al. (2011), surface complexation model parameters are independent of the experimental conditions (e.g., pH). Once parameterized with a set of batch equilibrium experiment data, SCMs are able to predict the nature of selectivity or competition in a multiple oxo-anion system in a wide range of conditions beyond those studied in limited isotherm tests.

Effects like changes in the influent concentration, pH, and EBCT on As breakthrough curves for a goethite-based GFH can be successfully predicted using a homogeneous surface diffusion model (HSDM) as an alternative to laborious column testings (Sperlich et al., 2005, 2008). The HSDM concept is based on the relationship between chemical equilibrium adsorption and kinetic mass transfer (Crittenden and Weber, 1978). In fact, the solute concentration gradient between the bulk solution and adsorbent surface creates a driving force across the stagnant film surrounding an adsorbent grain, and in particular within the tortuous pores of high surface area adsorbents such as GFH. An HSDM is a dual-resistance adsorption model that includes these effects of both mass transfer onto the adsorbent (surface film mass transfer) and into the adsorbent grains (intra-particle mass transfer). The textbook by Worch (2012) describes the HSDM concept and various practical applications relating to water treatment. The HSDM approach is by far superior to simple second order kinetic models. Once parameterized, it can be applied to predict adsorption kinetics across a range of solution conditions or presence of competing background solutes for akaganéite-based FBAs, which are some of the most frequently used technologies for removing target toxic oxo-anions worldwide. Kanematsu et al. (2012) first proposed that RSSCT results could be simulated if to allow the arsenate adsorption isotherms to be predicted using a simplified SCM. For the first time, we will show here that an advanced CD-MUSIC model can be used to predict the lifetimes of FBAs used to treat V-enriched groundwater with complex chemical background composition. In following this approach, we provide a sound basis for reliable HSDM model parameterization allowing the prediction of akaganéite-based FBA lifetimes for V-enriched groundwater with different chemical compositions. The aim of the present study was to extend the approach to simulating the combined effects of all solutes potentially affecting the lifetime of akaganéite-based FBA used to remove vanadate from groundwater. A coupled surface complexation model**−**HSDM model was developed and verified using data from pilot-scale FBA columns in real but anonymized German waterworks.

## 2 Experimental section

### 2.1 Characterization of the GFH adsorption efficiency

The akaganéite-based GFH used in this study was a commercial product GEH®102 produced by GEH Wasserchemie (Osnabrück, Germany) with product quality according to EN DIN 15029 and sold for use in FBAs worldwide (Driehaus et al., 1998). The fresh granular GFH was subjected to sieving analysis. The grain size range was 0.2–2.0 mm, and the median grain size was 0.7 mm (Fig. 1S in the Supporting Information). The GFH contained 45% ± 5% akaganéite and 55% ± 5% ferrihydrite determined by Rietveld analysis (Kersten et al., 2014). The nanoparticulate ferric hydroxide precipitate was granulated by a freeze/thaw procedure. This procedure gave a material with an inner microporosity of about 80%, a pore size distribution of 2–10 nm (Kumar et al., 2019), and a residual porewater content of about 45 ± 5 wt.-%. While the mineral density determined by gas pycnometer using He as the measuring gas (DIN 51913) was 3.67 g cm^−3^, the bulk density determined by water pycnometry was only 1.6 g cm^−3^ (Saha et al., 2005; Streat et al., 2008; Kersten et al., 2014). The GFH was not dried, but it was gently disaggregated in an agate mortar and pestle before use in batch adsorption equilibrium experiments. The material was not subjected to milling or heating, which might have caused ageing and phase transformation (Barrios et al., 1986). The specific surface area determined using the classical N_2_ BET method after freeze drying the material was 300 ± 30 m^2^ g^−1^.

Adsorption equilibrium experiments using the GFH were preferred using a slow completely mixed small batch reactor approach allowing the experiments to be performed in triplicate. Capped 50 mL low-density polyethylene centrifuge tubes were filled with freshly-boiled deionized high-purity water. Each stock reagent solution was freshly prepared from deionized water acidified to pH 4 and bubbled CO_2_-free using humidified Ar gas. Technical grade Ar was chosen because it is virtually free of O_2_ and CO_2_, whereas even pure nitrogen gas contains traces of O_2_ and CO_2_. Moreover, Ar is heavier than air, which enables an easy draining of air from bottles. GFH background electrolyte stock solutions containing 0.01, 0.05, and 0.1 M NaNO_3_ (Merck p.a. quality) were adjusted to pH 3 by adding conc. HNO_3_ (Merck Suprapure quality). Stock 0.1 M NaOH (Titrisol, Merck) solutions were prepared in NaNO_3_ solutions of respective molarity in CO_2_-free water. A 40 mL aliquot of the background electrolyte solution was added to each test tube, and sodium orthovanadate (Na_3_VO_4_, Sigma-Aldrich) was added as adsorbate. Then an amount of GFH was added to give a concentration in the solution of 1 g dry weight L^−1^. To each tube in a series of tests with the same background electrolyte concentrations, but different adsorbate concentrations, the NaOH solution was added dropwise to give a series of pH < 11. Each series contained tests at three different total adsorbate concentrations and three different ionic strengths.

The tubes were shaken horizontally (200 rpm) for 48 h at 25 ± 3 °C, then the final pH of the suspension and the residual dissolved ion concentrations in each tube were determined. The shaking time was long enough for equilibrium between the solid and solution to be achieved (Banerjee et al., 2008). A standard combination cell was not used to make the pH measurements. Instead, a Thermo Scientific Orion 9101BN/900200 half-cell pH probe with a separate KNO_3_ double junction reference electrode (Thermo Fisher Scientific, Waltham, MA, USA) was used to avoid interference by Cl^−^ ion leaching (Kersten et al., 2014). The electrode couple was calibrated using three commercial pH buffers (CertiPur, Merck) and the electrode readings were obtained using a stability criterion of <0.1 mV min^−1^. This, together with the linearity criterion, gave an overall uncertainty of pH 0.02. Each tube was then centrifuged at 5000 g for 30 min, then the supernatant was passed through a single-use 0.2-μm cellulose nitrate membrane syringe filter. The first few millilitres to pass through it were discarded for conditioning the membranes. Except for phosphate and silicic acid adsorbate tests (as described in Supporting Information), each filtered sample was acidified and stored at 4 °C until analysis. The residual solute concentrations were determined by an Agilent 7700 inductively coupled plasma mass spectrometer (Agilent Technologies, Santa Clara, CA, USA), which had a limit of detection for V of 1 μg L^−1^. The precision and accuracy of the analytical procedure were assessed by analysing a water reference material (NIST 1643e) with a certified V concentration. The batch equilibrium adsorption experiments using phosphate and silicic acid are described in detail in the Supporting Information.

### 2.2 Adsorption modelling

Models of surface complexation like the CD-MUSIC used here require a number of fixed parameters (including specific surface area, reactive site density, and adsorbent surface charge), as well as proton, electrolyte, and adsorbate ion equilibrium constants as discussed in detail in the Supporting Information. The PEST optimization code coupled to the geochemical speciation code Visual MINTEQ 3.1 was used to fit the data and parameterize the models (Gustafsson, 2018). The laborious CD-MUSIC modelling exercises performed in this and our previous studies ultimately allowed a thermodynamic data set to be constructed for 15 different surface complexes (Table 1), including those for phosphate and silicic acid as described in the Supporting Information. This dataset enabled competing effects between all major and toxic oxo-anions onto the akaganéite-based GFH to be predicted at any pH. This was because GFH adsorbs all oxo-anions at the same surface binding sites. This extended set of CD-MUSIC model parameters allows competing effect model scenarios to be set up, as demonstrated here for competition between vanadate and arsenate, silicic acid, and phosphate all together.

**Table 1:** Tableau defining the reactions involved in the formation of all the surface species studied using the 1 p*K* CD-MUSIC triple layer adsorption model of GFH. The density of singly coordinated surface groups ≡FeOH^−0.5^ was 6.1 sites nm^−2^, the density of triply coordinated surface groups ≡Fe_3_O^−0.5^ was 5.3 sites nm^−2^, the BET surface area was 300 m^2^ g^−1^, the Stern layer capacities C_1_ and C_2_ were = 0.74 and 0.93 F m^−2^, respectively, and the pH_PZC_ was 8.2 (Kersten et al., 2014).

Species	≡SOH^−0.5^, ≡S_3_O^−0.5^	Charge	Δ*z*_0_	Δ*z*_1_	Δ*z*_2_	H^+^	Na^+^	NO_3^−^_	Cl^−^	H_4_SiO_4_	PO_4_^-^^3^	AsO_4_^-^^3^	HVO_4_^-^^2^	log*K*
≡SOH_2_, ≡S_3_OH	1	0.5	1	0	0	1	0	0	0	0	0	0	0	8.2
≡SOH–Na, ≡S_3_O–Na	1	0.5	0	1	0	0	1	0	0	0	0	0	0	−0.6
≡SOH_2_–NO_3_, ≡S_3_OH–NO_3_	1	−0.5	1	−1	0	1	0	1	0	0	0	0	0	7.5
≡SOH_2_–Cl, ≡S_3_OH–Cl	1	−0.5	1	−1	0	1	0	0	1	0	0	0	0	7.7
≡(SO)_2_-PO_2_	2	−2	0.46	−1.46	0	2	0	0	0	0	1	0	0	28.1
≡(SO)_2_–POOH	2	−1	0.66	−0.66	0	3	0	0	0	0	1	0	0	32.9
≡(SO)_2_–Si(OH)_2_	2	−1	0.48	−0.48	0	0	0	0	0	1	0	0	0	4.8
≡(SO)_2_–Si_3_O_2_(OH)_5_O	2	−2	0.31	−1.31	0	−1	0	0	0	3	0	0	0	4.6
≡(SO)_2_–Si_4_O_3_(OH)_7_O	2	−2	0.33	−1.33	0	−1	0	0	0	4	0	0	0	8.3
≡(SO)_2_–AsO_2_	2	−2	0.47	−1.47	0	2	0	0	0	0	0	1	0	27.1
≡(SO)_2_–AsOOH	2	−1	0.58	−0.58	0	3	0	0	0	0	0	1	0	31.3
≡(SO)_2_–AsOH	2	−1	0.30	−0.30	0	0	0	0	0	0	0	0	0	6.05
≡(SO)_2_–V(OH)_2_	2	0	0.9	0.1	0	3	0	0	0	0	0	0	1	27.4
≡(SO)_2_–VOOH	2	−1	0.9	−0.9	0	2	0	0	0	0	0	0	1	24.5
≡SO-VO_2_	1	−1.5	0.9	−1.9	0	1	0	0	0	0	0	0	1	14.0

### 2.3 Adsorbent column test experiments

RSCCT experiments were performed using laboratory-scale columns with the GFH, but of original grain size distribution thus deviating somewhat from the original RSCCT concept by Westerhoff et al. (2005). Each column had an inner diameter of 25 mm and a length of 200 mm, and contained 65 g of GFH, giving a bed depth of 105 mm. The inflow rate was 16 mL min^−1^ resulting in an EBCT of 3.2 min. The hydraulic parameters are compiled in Table 1. The influent was Milli-Q water adjusted to pH 7.5 and contained 10 μmol L^−1^ each of arsenate, phosphate, and vanadate. Each experiment was performed until 50,000 BVs had been treated. Effluent samples taken at time intervals were passed through disposable 0.2-μm cellulose nitrate membrane syringe filters. The first few millilitres to pass through the filter were discarded, and the remaining filtrate was analysed directly (for P) or acidified by adding 0.1% analytical reagent grade HCl before analysis for As and V. The As(V) and V(V) concentrations in the effluent were determined using an Agilent 7700 inductively coupled plasma mass spectrometer following standard methods (DIN EN ISO 17294-2), and the phosphate concentrations were determined using a Methrohm 790 ion chromatograph.

Pilot-scale adsorbent column tests were performed at three German groundwater treatment works, denoted in an anonymized way “K”, “W”, and “P”. High V concentrations of up to 50 μg L^−1^ have been found in well waters treated at these waterworks (Bahr et al., 2019). The aquifers supplying the raw water contain V-bearing fluorapatite (as geogenic source for V in many volcanic rocks; Härter et al., 2020) that leaches V(V) to the groundwater exceeding the threshold values. The phosphate concentrations in the waters are therefore also high, and the molar P/V ratio of about 10 is the same as that of the solid V-bearing fluorapatite phase indicating stoichiometric dissolution. Hydrogeochemical modelling using PhreePlot indicates that vanadate(V) species dominate the V-enriched water (Härter et al., 2020). However, the As concentrations were low (<3 μg L^−1^) in these oxic waters, meaning that As does not need to be removed at these sites. The V concentrations were correlated with the silicic acid concentrations (which were also high, up to 40 mg L^−1^) due to dissolution of amorphous glass phases in the volcanic aquifer rocks (Table 2). Each FBA test column was filled with 80–100 kg of GFH, and the columns were used in bypass pipes beside the main water treatment lines. At “K” and “W” waterworks, FBA columns with inner diameters of 300 mm were used, but at “P”, a wider column of a pool filter type with a diameter of 500 mm was used. The design parameters for the pilot FBAs are compiled in Table 2. The inflow and outflow water compositions were monitored each week for a minimum of 1 y using the analytical methods described above.

**Table 2:** Parameters for the RCSST test and the three pilot scale FBAs.

**Parameter**	**Unit**	**RCSST**	**Waterworks (pilot scale)**		
			“K″	“W″	“P″
Diameter	mm	25	300	300	500
Bed height	mm	105	1200	1200	360
Bed volume	L	0.052	87	77	70
Bed mass (wet-wt.)	kg	0.065	100	90	80
Bed density (wet)	kg/m^3^	1280	1150	1150	1150
Particle density (wet)	kg/m^3^	1590	1590	1590	1590
Mean particle diameter	mm	0.7	0.7	0.7	0.7
Flow rate	m^3^/h	9.6·10^−4^	0.52	0.96	1.4
EBCT	min	3.2	10	4.9	3.0
Final throughput	BV	50,000	60,000	50,000	260,000
pH	–	7.5	7.4	7.5	7.6
Mean vanadium	μg/L	510	48	42	19
influent concentration	VO_4_–V				
Mean phosphate	μg/L	310	700	560	200
influent concentration	PO_4_–P				
Mean arsenate	μg/L	750	<3	<3	<3
influent concentration	AsO_4_–As				
Mean silicic acid	mg/L	–	37	40	18
influent concentration	SiO_2_–Si				

## 3 Results and discussion

### 3.1 Surface complexation modelling of adsorbate competition effects

Dissolved V occurs in different coordination environments depending on pH (Gustafsson, 2019). In an aerobic solution, V is pentavalent and forms more- or less-protonated vanadate oxyanion species (H_2_VO_4^−^_ or HVO_4_^−^^2^) at pH values > 4 (Fig. S3, Supporting Information). However, at low pH values (pH < 4), the pentavalent V is present as the vanadyl cation VO_2_^+^ rather than orthovanadic acid H_3_VO_4_, as is the case for other oxo-anions. The thermodynamic stability constants for the protonation equilibria of the more than a dozen mononuclear and oligomer vanadate species were updated using data published by Gustafsson (2019). However, only mononuclear vanadate species were considered in the adsorption modelling exercise, because monomeric vanadate species are predominant at low V concentrations in groundwater. Oligomeric vanadate species may become important at V concentrations >0.1 mmol L^−1^ and at low pH (Gustafsson, 2019).

**Fig. 1:**
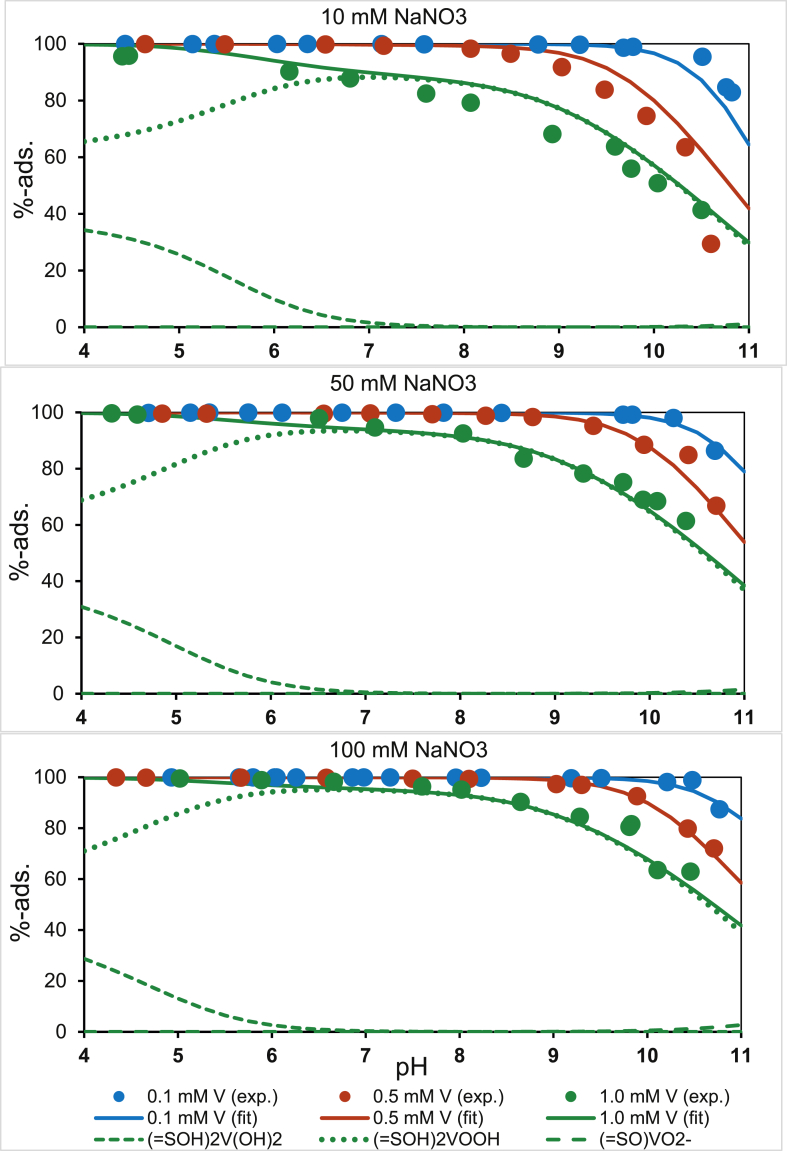
Plots of vanadate adsorption vs. pH for GFH. The dots represent experimental data and the lines show the model fits achieved using the CD-MUSIC parameters suggested by Larsson et al. (2017) and optimized for the GFH.

As is typical for oxo-anions, plots of the percentage vanadate adsorbed vs. pH indicated that maximum adsorption occurred in the acidic to circumneutral pH range, but the adsorption edge at low V concentrations was shifted until it reaches above pH > 10 (Fig. 1), meaning that vanadate is one of the oxo-anions that most strongly adsorbs to Fe oxyhydroxides. The adsorption edge shifted to less alkaline pH values as the V concentrations increased. Surface complex formation was analysed by synchrotron-based XANES, and it was confirmed that V(V) remained as tetrahedral vanadate and had not reduced or had its coordination changed by its adsorption to ferrihydrite, and EXAFS indicated that there were edge-sharing bidentate complexes of V(O,OH)_4_ tetrahedra with Fe(O,OH)_6_ octahedra on the ferrihydrite surfaces (Larsson et al., 2017). Larsson et al. (2017) used their spectroscopic results to constrain a CD-MUSIC adsorption model for V(V) surface complexes representing different degrees of protonation:

(1) 2FeOH^−0.5^ + 3H^+^ + HVO_4_^−^^2^ = (FeO)_2_V(OH)_2_^0^ + 2H_2_O

(2) 2FeOH^−0.5^ + 2H^+^ + HVO_4_^−^^2^ = (FeO)_2_VOOH^−1^ + 2H_2_O

(3) 2FeOH^−0.5^ + H^+^ + HVO_4_^−2^ = (FeO)_2_VO_2_^−2^ + 2H_2_O

Although the H_2_VO_4_^−^ species dominates at circumneutral pH conditions, the stoichiometric equations are formulated with the HVO_4_^−2^ species, since it is default input in the Visual Minteq code used for data fitting. The first two complexes dominate the surface speciation of vanadate across a wide pH range, but the third (least protonated) complex is important only at high pH for high surface coverage. In principle, vanadate could be re-formulated as a mono-dentate surface complex like other oxo-anions as discussed by Larsson et al. (2017):

(4) FeOH^−0.5^ + H_2_VO_4_^−^ = FeOVO_2_^−1.5^ + H_2_O

**Fig. 2:**
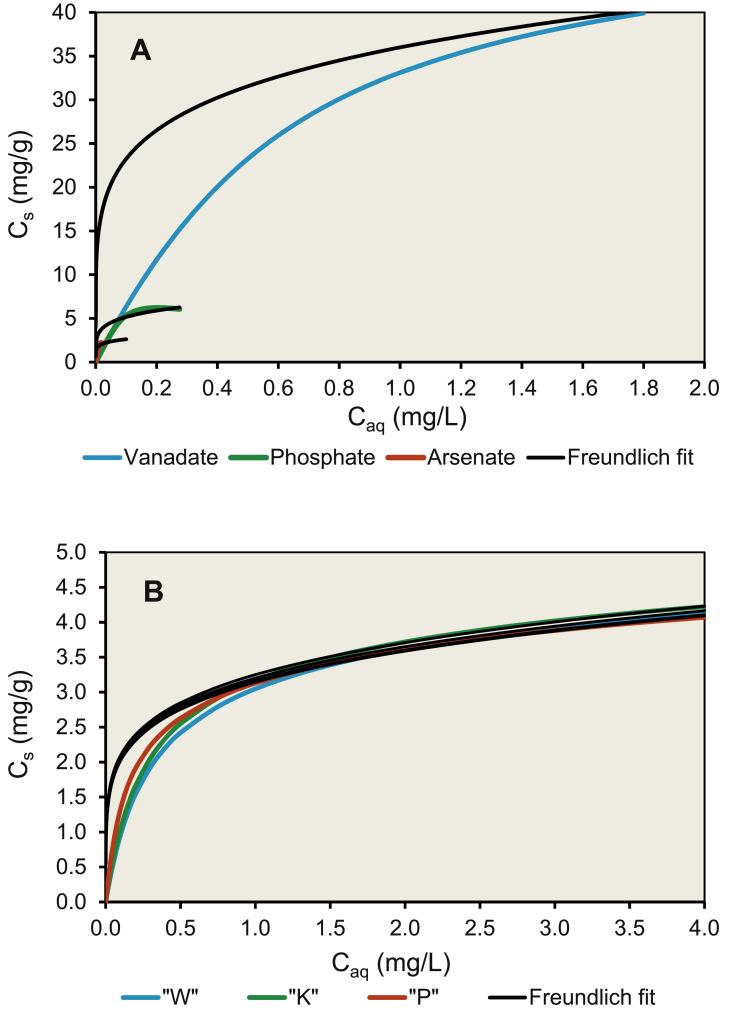
Equilibrium adsorption isotherms predicted using the CD-MUSIC model (coloured lines) for the influent water and the amount of GFH used in (A) the RCSST experiments, and (B) the pilot scale FBAs. The black lines show non-linear Freundlich isotherms fitted to the maximum saturation predicted by the CD-MUSIC model for the multi-solute competitive GFH adsorbent system. The slope and saturation non-linearity could not both be fitted by a Freundlich isotherm. The non-linearity was therefore represented by fixing the Freundlich exponent at *n* = 0.19, and the competitive saturation effect was represented by increasing the Freundlich constant in the order arsenate (*K*_*F*_ = 4) < phosphate (*K*_*F*_ = 8) < vanadate (*K*_*F*_ = 36).

However, this surface complex also does not play an important role. Our model was fitted using the surface complexation reactions (1) and (2) and was of good quality overall, as shown by the solid and dotted lines in Fig. 1. The fitted complexation constants for the GFH are compiled in Table 1. The CD coefficients were slightly altered when the charge in the zero plane became more positive because of decreasing protonation. This occurred through more of the negative charge of the vanadate complex being shifted into the first outer-sphere plane (Δ*z*_0_ = 0.9 v.u. and Δ*z*_1_ = −1.9 v.u. for the most protonated third complex). Larsson et al. (2017) suggested that the opposite charging behavior would occur. However, a final conclusion on this cannot be made without advanced molecular modelling. Nonetheless, the complexation constants that we derived were similar to those found by Larsson et al. (2017). Same approach was used to derive at CD-MUSIC data for phosphate and silicic acid (Figs. S4 and S5 in the Supporting Information). Published data on phosphate, silicic acid, and vanadate adsorption can favorably be re-fitted using our parameter set (Figs. S6–S8 in the Supporting Information). All these data result in an affinity sequence of H_2_VO_4_^−^ > H_4_SiO_4_^0^ > H_2_PO_4_^−^ > H_2_AsO_4_^−^ for the GFH adsorbent (Fig. S8 in the Supporting Information). If to quantify the selectivity for the target oxo-anion vanadate in comparison to the other competitive oxo-anions, a selectivity coefficient *β*_*t*_ = *K*_*d,t*_/*K*_*d,c*_ can be introduced, where *K*_*d,t*_ is the distribution coefficient of the target oxo-anion, and *K*_*d,c*_ is the distribution coefficient of the background oxo-anion (Pincus et al., 2020). Selectivity coefficient values of *β*_*t*_ = 1.2 for phosphate and 2.5 for arsenate calculated from the batch equilibrium data shown in Fig. 2 (with equal equilibrium solute concentration of 0.1 mg L^−1^) are all above unity and thus demonstrate high selectivity of the GFH towards the target oxo-anion vanadate. This selectivity can be understood on a theoretical basis if to recall the Pearson Hard Soft Acid Base (HSAB) principle (Pincus et al., 2020). According to this principle, the oxo-anions as hard Lewis bases (electron donors) are adsorbed by the iron hydroxide surface as a hard Lewis acid (electron acceptor). The selectivity varies with the relative Lewis base hardness, which can be assessed by the *pK*_*a*_ values of the deprotonation of the respective oxo-anion species and suggests the same ranking of *pK*_*a*_ = 8.75 (H_2_VO_4_^−^) > 7.20 (H_2_PO_4_^−^) ≈ 7.0 (H_2_AsO_4_^−^).

### 3.2 HSDM simulation of the RSSCT breakthrough curves

The HSDM approach used to model the BTCs was described in the textbook by Worch (2012). Several simplifying assumptions were made when developing the HSDM. These were that (i) adsorbent grains occur as spheres of homogeneous composition (the H in HSDM), (ii) both linear aqueous phase mass transfer and solid phase mass transfer occur only through surface diffusion (the SD in HSDM), (iii) plug-flow conditions occur in the adsorbent bed, (iv) the hydraulic loading rate and diffusion coefficients are constant, and (v) adsorption isotherms are used to describe the adsorption equilibrium. Three essential intrinsic dimensionless parameters of the HSDM (the Biot number *Bi*, the modified Stanton number *St*, and the solute distribution coefficient *D*_g_) define the shape of the BTC and are derived from in total 10 operational, equilibrium, and kinetic input parameters, as shown previously for arsenate removal by GFH adsorbent columns (Badruzzaman et al., 2004; Sperlich et al., 2008; Pérez-Foguet et al., 2013). The operational parameters include the adsorbent mass *m*, the filter bed porosity *e*_B_, the bulk density of the adsorbent grain particles including the inner porosity filled by water ρ_P_, the mean diameter of the adsorbent grains determined by sieve analysis *d*_P_, the aqueous influent concentration of the solute *C*_*0*_, and the constant volumetric flow rate through the adsorbent column *Q*. All these physical and chemical parameters can be measured for the RSSCT experiment and are compiled in Table 2. Aim of this research was to reduce the remaining free parameters for fitting of the BTCs to only one, which is the solute intra-particle diffusion coefficient or Biot number.

In our HSDM model, adsorbate competition was considered only with regard to the description of the multi-solute adsorption equilibrium, and the effects of this competition on mass transfer (and therefore the adsorption kinetics) were assumed at first approximation to be negligible, as also suggested by Worch (2012). The same single-solute mass transfer coefficients were therefore used for all of the oxo-anions at first approximation to predict the BTCs for the multi-solute systems. In this respect, the method used to estimate the HSDM model parameters was not different from those used previously to estimate parameters for a single solute arsenate or phosphate system (Sperlich et al., 2005, 2008). The fixed-bed adsorption simulation tool FAST v.2.1 (www.fast-software.de; typical screenshot shown in Fig. S9 of the Supporting Information) was used to perform the HSDM simulations. A simulation iteration typically took a few min when run as four parallel threads on a laptop. The bed density and the EBCT were calculated using this software code on basis of the hydrodynamic parameters chosen for the experiments (Table 2). Further kinetic HSDM model parameters include the film diffusion coefficient *k*_L_, and the surface diffusion coefficient *D*_S_.

The film diffusion coefficient influences the slope at beginning of the BTC curve (Worch, 2012). The diffusion coefficient for all three oxo-anions was fixed at *k*_L_ = 2.5·10^−6^ m s^−1^ as first guess. This is reasonable because the film diffusion depends on the advective flow rate more than on the solute, and the flow rate will be the same for all oxo-anions. A Stanton number *St* of between 1.1 and 3.0, as the ratio between the rate of transport by film mass transfer and the measured rate of transport by advection used in this study (and also by Pérez-Foguet et al., 2013), indicates that film mass transfer is slightly faster than advection and therefore not rate limiting. This appears reasonable for the relatively fast inflow rate of *a*_*VR*_ = 2–7 m h^−1^ applied during the RSSCT and pilot scale column tests. The intra-particle diffusion coefficient *D*_*s*_, on the other hand, strongly depends on the characteristics of the adsorbate and is between 10^−16^ and 10^−14^ m^2^ s^−1^ (Badruzzaman et al., 2004; Sperlich et al., 2005, 2008). The surface diffusion coefficient may be estimated experimentally for each single adsorbent system by using a differential column batch reactor (DCBR) test, or used as fit parameter in a multi-solute system (Worch, 2012).

Adsorption in the HSDM is commonly described by the Freundlich adsorption isotherm, *C*_*ads*_ = *K*_*F*_*·C*_*0*_^*n*^. Freundlich coefficients are often used as free fitting parameters in HSDM systems. For example, Sperlich et al. (2008) suggested that strongly non-linear Freundlich exponents of *n* = 0.19 should be used for arsenate and phosphate, which we adopted here for vanadate. The non-linearity mimics the limited adsorption capacity that is a necessary pre-condition for constructing a BTC. The Freundlich constants were used to differentiate between adsorption affinities of the solutes. However, the Freundlich constant for arsenate was found to be double that of phosphate by Sperlich et al. (2008), which contradicts our general understanding of affinity series in the adsorption of oxo-anions by iron hydroxides. Just the opposite should be the case, as shown in Fig. 2 and also by the increase of the solute distribution coefficient *D*_*g*_ in the order arsenate – phosphate – vanadate (Table 3).

**Table 3:** HSDM parameters for the RSSCT experiments and the three pilot-scale FBAs.

**Parameter**	**Unit**	**RSSCT**	**Waterworks**		
			“K”	“W”	“P”
Freundlich constant *K*_*F*_ (arsenate)	L/g	4	–	–	–
Freundlich constant *K*_*F*_ (phosphate)	L/g	8	–	–	–
Freundlich constant *K*_*F*_ (vanadate)	L/g	36	3.2	3.2	3.2
Freundlich exponent *n* (all oxyanions)	–	0.19	0.19	0.19	0.19
Film diffusion coefficient *k*_*L*_	m/s	2.5·10^−6^	2.5·10^−6^	2.5·10^−6^	5.0·10^−6^
Surface diffusion coefficient *D*_*s*_	m^2^/s				
Arsenate		2·10^−15^	–	–	–
Phosphate		4·10^−16^	–	–	–
Vanadate		1·10^−16^	1·10^−16^	1·10^−16^	1·10^−16^
Solute distribution *Dg*	–				
Arsenate		4.6·10^4^	–	–	–
Phosphate		1.3·10^5^	–	–	–
Vanadate		4.1·10^5^	5.8·10^5^	6.2·10^5^	1.2·10^6^
Biot number *Bi*	–				
Arsenate		39	–	–	–
Phosphate		67	–	–	–
Vanadate		89	37	37	37
Stanton number *St*	–	1.10	2.97	1.56	1.86

The CD-MUSIC model can be used to study competitive effects on the adsorption equilibrium. The model can be used to predict Freundlich adsorption isotherm parameters, but not a Langmuir adsorption isotherm model due to its limitation of equal activity of the various V surface complexes. The key parameter for a competitive multi-solute adsorbate system with a strong adsorbent is the surface saturation effect. An adsorbate with a lower affinity tends to be outcompeted by (and therefore has a lower saturation level than) an adsorbate with a higher affinity. To model these data, the adsorption isotherms were first simulated using our CD-MUSIC model. For this the equal inflow concentrations for all three adsorbates, the adsorbent amounts in the test columns (65.4 g wet-weight or 37.3 g dry-weight), and volume of water treated during the whole RCSST experiment run (50,000 times 51.5 mL BV = 2500 L; Table 2) were used. As expected for a strong adsorbent (high solute distribution coefficient *D*_*g*_ in the order of 10^5^; Table 3), the GFH adsorbed almost 100% of all three oxo-anions at low concentrations. The isotherm slope at low oxyanion concentrations was therefore the same for all the oxo-anions and was determined only by the amount of adsorbent present for the total water volume treated.

**Fig. 3:**
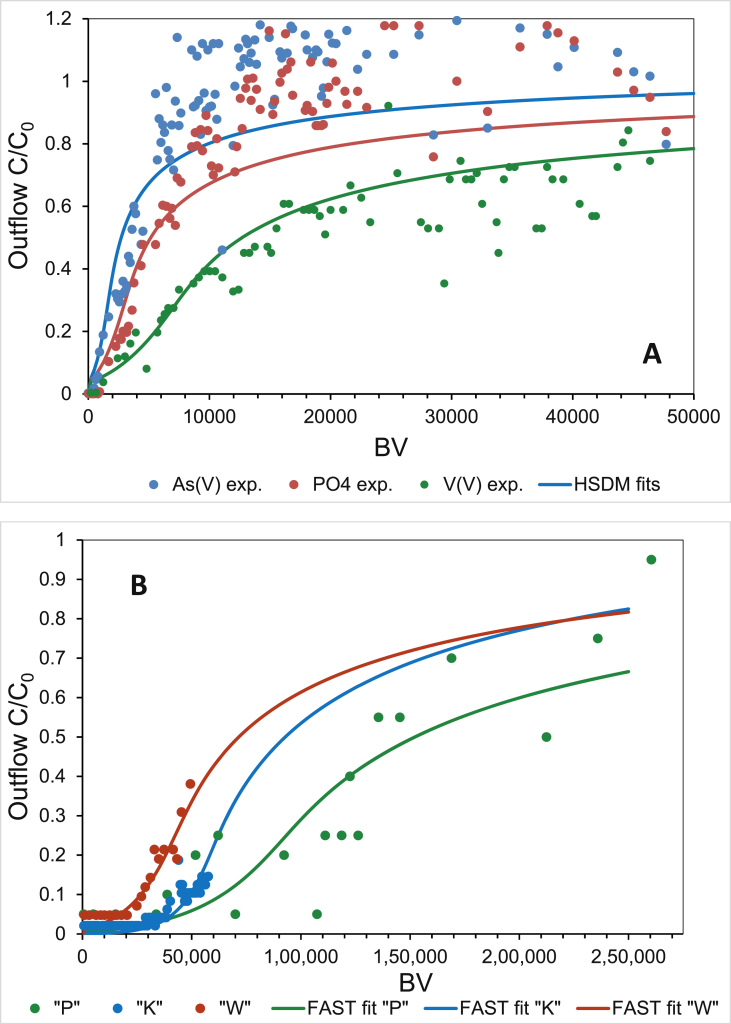
Breakthrough curves for (A) the RCSST experiments, and (B) the pilot scale FBAs (the dots represent experimental data), with lines fitted using the HSDM option of the FAST v.2.1 software code with the parameters compiled in Table 2, Table 3.

At higher concentrations, however, the oxo-anion with highest affinity (vanadate in this case) may outcompete an oxo-anion with lower affinity (phosphate, silicic acid, and arsenate, in that order) and therefore limit the adsorbent capacity for the latter adsorbates. The Freundlich isotherms must therefore be strongly non-linear, with Freundlich exponent *n* < 1, to represent the quite different adsorption limits in a multi-solute system predicted by the CD-MUSIC model. The exponent was therefore fixed at *n* = 0.19 for all three oxo-anions as suggested by Sperlich et al. (2008). As shown in Fig. 2a, the slope of the curves could not well be reproduced by the non-linear Freundlich isotherms. However, the different degrees of saturation in the competitive multi-component system could well be represented by the Freundlich constants thus predicted. They increased in the order arsenate (*K*_*F*_ = 4) < phosphate (*K*_*F*_ = 8) < vanadate (*K*_*F*_ = 36) according to the order in which the adsorption affinity increased in the competitive multi-solute system. Since FBA performance is a combination of adsorption capacity and adsorption kinetics, the Freundlich constants describing adsorption capacity rather than the exponents describing the slope are key parameters for modelling of the BTCs by the HSDM approach (Sperlich et al., 2008; Worch, 2012). The thus fixed HSDM parameters (Table 2, Table 3) enabled BTCs for arsenate, phosphate, and vanadate to be fitted to the RSSCT data (Fig. 3a).

Sperlich et al. (2008) and Pérez-Foguet et al. (2013) did not report film mass-transfer rate numbers, but surface diffusion constants and Biot numbers only. Pérez-Foguet et al. (2013) pointed out that film mass-transfer dominates when *Bi* < 10^0^ and surface diffusion is dominant when *Bi* > 10^2^. Hence, the higher the Biot number *Bi*, the higher is the rate of film diffusion in comparison to surface diffusion. If *Bi* > 50, the influence of the film diffusion on the overall adsorption rate becomes negligible (Worch, 2012). In our study, the best-fit *Bi* numbers were in the range 40–90 (Table 3), so surface diffusion must influence the BTCs the most. Relatively slow and therefore rate limiting intra-particle mass transfer (*D*_*s*_ = 1·10^−16^ m^2^ s^−1^ or 1·10^−12^ cm^2^ s^−1^) caused the BTC slope to be smoother for vanadate than for arsenate and phosphate (Fig. 3a). Element maps of GFH grains indicated that there was an adsorbate distribution gradient throughout the grain interior even after use for 2 y as a FBA in a waterworks (Fig. S2 in the Supporting Information).

Fig. 3a shows also that a displacement process takes place, leading to a quite different breakthrough behaviour of the three oxo-anions. As a typical result of competition and displacement, a slight concentration overshoot could be observed for the two weaker absorbed oxo-anions arsenate and phosphate. This effect has not yet been reported in literature, but can in principle be explained by their different traveling velocities within the adsorbent column (Worch, 2012). Since the traveling velocity depends on the adsorption affinity of the solute, the weaker absorbed oxo-anion travels faster through the adsorbent column, while the stronger adsorbed oxo-anion lacks behind. Later, when the stronger adsorbed oxo-anion reaches the same layers, a new equilibrium state is established resulting in partial displacement of the previously adsorbed oxo-anion of weaker affinity. As a result of this displacement process, the concentration of the latter component becomes higher than its initial inflow concentration. Except of the vanadate with highest adsorption affinity, the other two oxo-anions are obviously subject to this displacement process and hence concentration overshoot, whereby the weaker adsorbed arsenate shows the overshoot effect first, followed by the phosphate a bit later (Fig. 3a). Unfortunately, this overshoot effect cannot be simulated by a model BTC, which must run asymptotically towards *C*/*C*_*0*_ = 1. Because of the high initial concentrations of about 10 μmol L^−1^, the overshoot effect leads to an extensive plateau at about *C*/*C*_*0*_ = 1.2 in between 10,000 and 40,000 BVs, and levels out after about 40,000 BVs towards *C*/*C*_*0*_ = 1 (Fig. 3a).

### 3.3 HSDM simulation of pilot-scale column BTCs for the three water treatment works

The water supplied to water treatment works “K” and “W” had similar and relatively high phosphate and silicic acid concentrations, and also similar V concentrations, but more than twice as high than inflow concentrations in waterworks “P” (Table 2). It has previously been found that elevated phosphate and vanadate concentrations in groundwater of the study area are of geogenic source (Härter et al., 2020). The As concentrations in the oxic waters supplied to all three waterworks were relatively low (<3 μg L^−1^). Arsenate therefore did not compete with vanadate, but phosphate with by one order of magnitude higher concentrations may eventually compete with vanadate. The vanadate BTCs for the pilot test columns in the three waterworks are shown in Fig. 3b. The BTCs were asymptotic, so accurate breakthrough times could not be determined. From a theoretical point of view, the time at C/C_0_ = 0.05 is conventionally used as the breakthrough point (Worch, 2012), but in practice the time at which the concentration exceeds the relevant regulatory value (4 μg L^−1^ for vanadate) is of most interest. All three BTCs followed typical trends, the outflow V concentration being below the limit of detection (1 μg L^−1^) at the beginning, and then slowly but steadily increasing until the threshold was exceeded at about 30,000 BVs at location “W”, 40,000 BVs at location “K”, and 110,000 BVs at location “P”. The FBA service lifetime would therefore be reached at 16 months at “K”, at 7 months at “P”, and only 3 months at “W”. This rather different lifetimes clearly warrant further explanation. There is a huge scatter in data for “P” site which also warrants explanation. First of all, the sampling intervals were a bit excessively long. Therefore, the data density was not as good as for the other two sites. Second reason was the relatively low bed height of only 36 cm. Even though the design parameters were correct (EBCT and flow rate), the relatively low bed height in the pool filter means that the filter bed is more susceptible to short-circuit effects. If the bed was blocked somewhat by the introduction of turbid substances from the raw water, preferred flow channels were formed and an earlier breakthrough occurred. If the filter is then backwashed, the flow conditions became more even and the adsorption capacity improved again. This is a phenomenon that can sometimes be observed in practice also with large FBA systems. In the case of the pilot filter at site “P”, glitches in the BTC at approximately 60,000 BV and at 80,000 BV can be attributed to this backwashing effect.

All of the water samples were in a similar pH range of 7.4–7.6 (Table 2). It appears that the different groundwater chemistries at the three selected waterworks affect the BTCs and hence the lifetime of the FBA test columns. However, the situation becomes more complex considering the CD-MUSIC predictions for the Freundlich adsorption isotherms for all three sites with their different chemical compositions (Fig. 2b). First of all, the different concentrations of the co-adsorbing solutes like phosphate and silicic acid do obviously not play a role, because the Freundlich coefficients predicted for vanadate by the CD-MUSIC model are the same at all three sites (*K*_*F*_ = 3.2 ± 0.05 for *n* = 0.19). The V concentration in the influent water at site “P” was just half those in the influents to the other two waterworks, and this clearly renders the processing capacity at “P” higher than if it received the same water as the other works, in spite of the lowest EBCT chosen (Table 2). However, the rather different lifetimes for “K” and “W”, which received water with similar V concentrations, cannot as easily be explained from a chemical point of view. The similar co-solute concentrations do obviously not play a role. The difference in capacity can rather be attributed to the physical difference in dimension of the columns used, which gave an EBCT twice higher and therefore more favourable adsorption conditions for “K” than for “W”. The twice longer EBCT (lower flow rate) for “K” than for “W” clearly caused the vanadate removal in the presence of competing ions to be much more successful. Furthermore, the adsorbent bed was not homogeneously loaded with V. Chemical analysis of the adsorbent material after the end of the test runs at site “P” indicated a vertical concentration gradient, and that the maximum V loading was 7.5 mg g^−1^ dry-weight in the topmost bed horizon and the minimum V loading was 2 mg g^−1^ dry-weight in the bottommost bed horizon (Bahr et al., 2019), with an average of 4 mg g^−1^ dry-weight just as predicted by the CD-MUSIC model (Fig. 2b).

## 4 Conclusion

The effects of the phosphate and silicic acid concentration, pH, and EBCT on V(V) breakthrough curves were predicted using the HSDM approach. The Freundlich adsorption isotherm data required for the HSDM were predicted by the CD-MUSIC surface complexation model. It was set up by adsorption equilibrium data for all oxo-anions, and then applied on the compositions of the influent water of three different water treatment works. The pilot test column BTCs for all three water treatment works were predicted well using a unique Freundlich constant derived on basis of the multi-solute CD-MUSIC model, a single surface diffusion coefficient derived from the RSSCT column test, and therefore the same Biot number fixed at *Bi* = 37 for all three sites. The coupled model approach was thereby demonstrated to predict the individual effects of these parameters on oxo-anion removal performance of the GFH. It was also shown that GFH removal performance for vanadate is not much influenced by arsenate, phosphate, and silicic acid contents of the groundwater, whereas vanadate may lead to a BTC overshoot for the other oxo-anions. The FBA lifetimes for removal of the vanadate are therefore controlled by operational parameters such as the V concentration in the effluent waters and the EBCTs rather than the groundwater chemistries. Moreover, our combined equilibrium and kinetic model approach allows any combinations of environmental conditions to be used in scenario models to predict lifetimes of the adsorbent columns. The approach demonstrated here can be extended to include other relevant chemicals in water, such as dissolved uranium or even organic chemicals, provided that the relevant CD-MUSIC model parameters are available. The approach is suitable for predicting equilibrium parameters for HSDM models, and reduces the fitting to the kinetic parameters determined by the individual column design. The large number of interferences that have to be studied imply that such a modelling approach could help to optimize the designs of factorial experiments to identify the most important interferences and to minimize the number of test columns required.

## Declaration of competing interest

The authors declare that they have no known competing financial interests or personal relationships that could have appeared to influence the work reported in this paper.
