# Supplementary material for: Predicting breakthrough of vanadium in fixed-bed absorbent columns with complex groundwater chemistries: A multi-component granular ferric hydroxide−vanadate−arsenate−phosphate−silicic acid system
Source: Water Res X. 2020 Aug 5;9:100061. doi: 10.1016/j.wroa.2020.100061 (PMC7426449; doi:10.1016/j.wroa.2020.100061)
Supplement: Multimedia component 1 [file mmc1.pdf]

Supporting Information

Predicting breakthrough of vanadium in fixed-bed absorbent columns with complex groundwater chemistries: a multi-component granular ferric hydroxide–vanadate–arsenate–phosphate–silicic acid system

Anna Dabizha[1], Carsten Bahr[2], Michael Kersten[1,]*

[1]Geosciences Institute, Johannes Gutenberg University, Becherweg 21, Mainz 55099, Germany

[2]GEH Wasserchemie GmbH & Co. KG, Adolf-Köhne-Straße 4, Osnabrück 49090, Germany

*Corresponding Author E-mail address: kersten@uni-mainz.de

12 pages, 9 figures

1. GFH adsorbent characterization p. S2
2. V speciation in effluent water of waterworks “K” p. S3
3. Surface complexation model parameterization
3.1. CD-MUSIC model p. S3
3.2. Arsenic p. S4
3.3. Phosphate p. S4
3.4. Silicic acid p. S6
4. Published data re-fitted using the CD-MUSIC model p. S8
5. Screenshot of the FAST v.2.1 software code p. S10
References p. S10

1. GFH adsorbent characterization

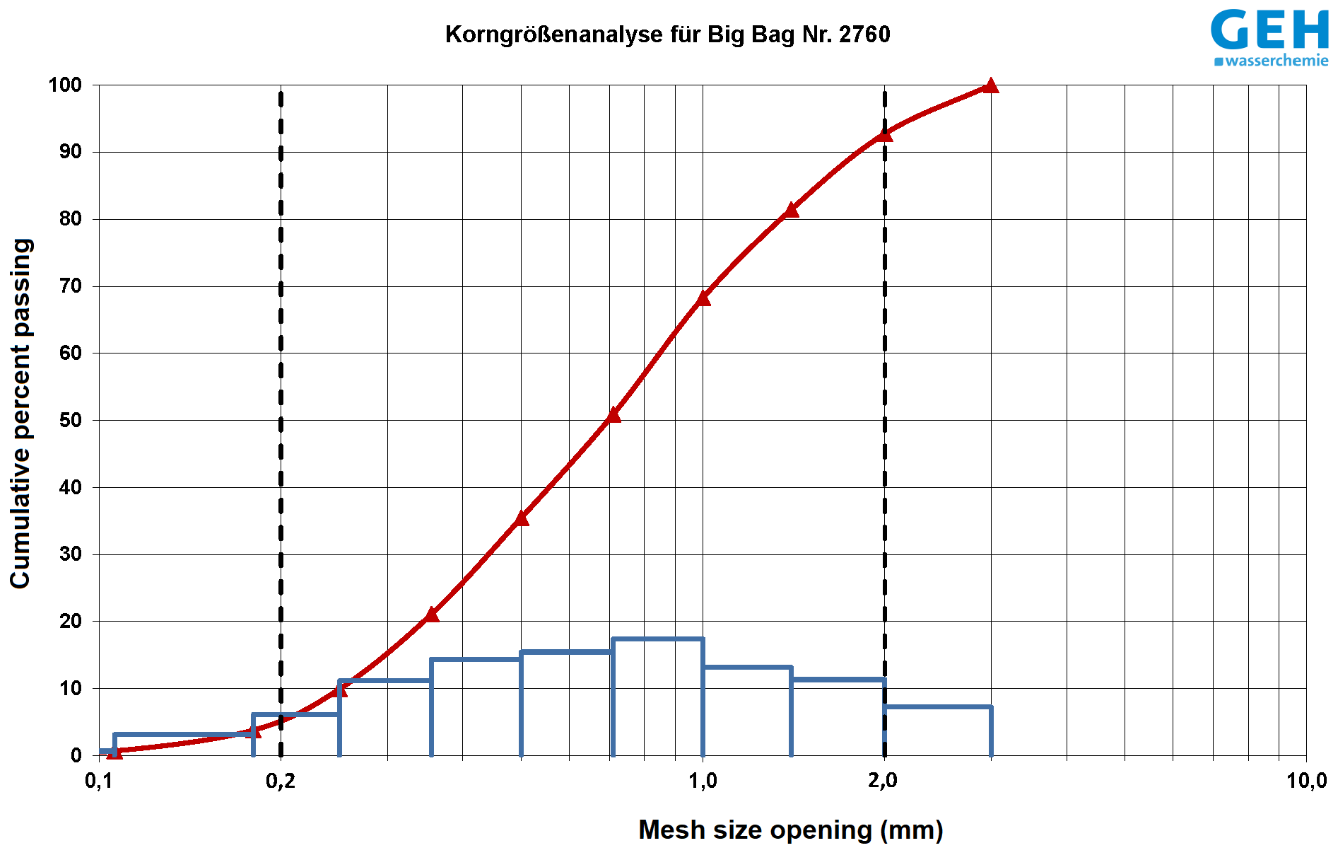

Figure S1: Sieving analysis results. Sieving analysis was performed according to the DIN EN ISO 15029 method on a representative GFH sample to estimate the mean grain size (d_{50} = 0.70 mm). The results gave a uniformity coefficient C_U of d_{60}/d_{10} = 3.5, calculated as described in the DIN EN ISO 14688-2:2004 method.

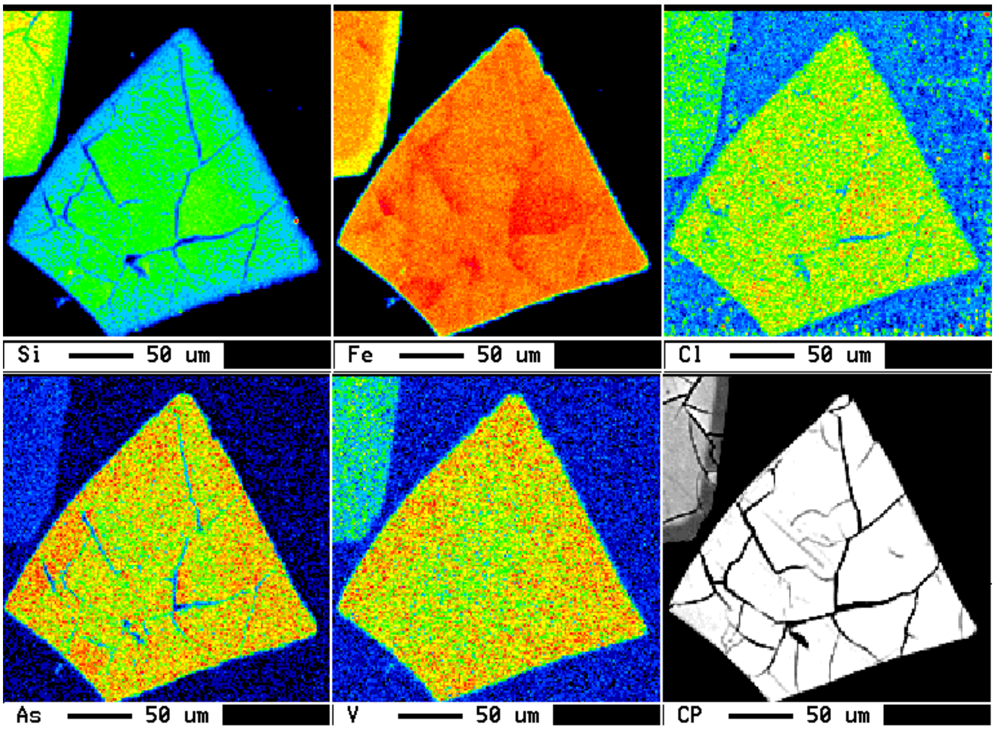

Figure S2: Element map of a GFH grain using an electron probe microanalysis (the diameter can be estimated using the 50 µm scale bar shown below each image). The GFH grain has been used for 2 y in a waterworks FBA column. Vanadium (middle of the lower row) had a concentration gradient, with lower concentrations in the middle of the grain indicating very slow intra-particle diffusion of the oxyanion.

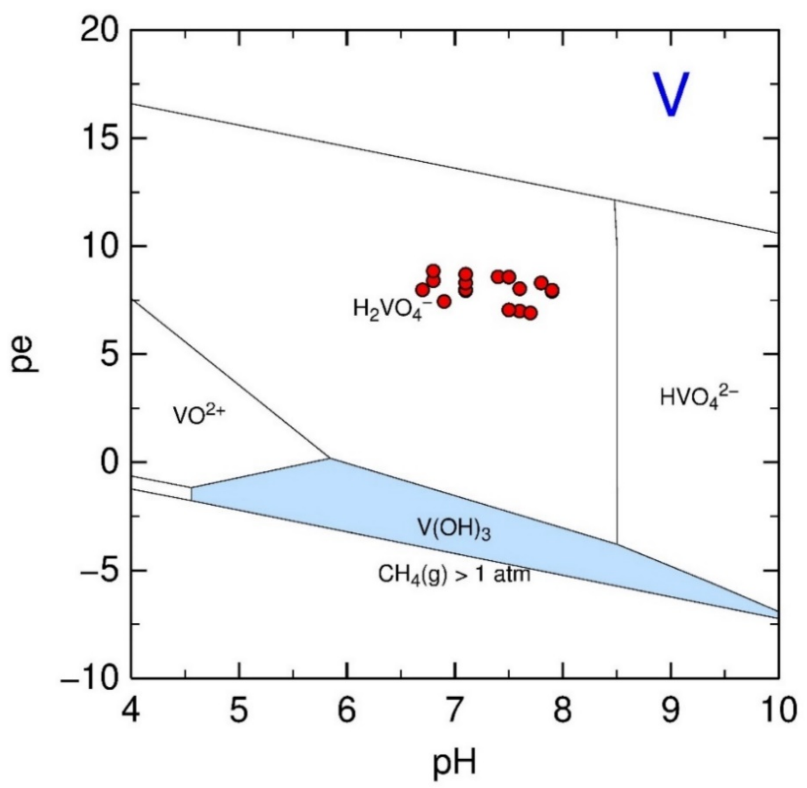

Figure S3: A pe-pH diagram of the V speciation predominance for spring and well water samples at waterworks "K". The dissolved V(IV) and V(V) species are represented by the white fields, the sparingly soluble V(III) hydroxide marks the lower bound with the blue domain. The red dots mark the pe/pH distribution range of the water samples. The diagram was prepared using the USGS code PhreePlot (Kinniburgh and Cooper, 2011) with thermodynamic constants compiled by Gustafsson (2019).

2. Surface Complexation Model Parameterization

2.1. The CD-MUSIC model

The CD-MUSIC is a state-of-the-art model for describing oxyanion adsorption to single oxyhydroxide surfaces (Hiemstra et al., 2006). The model is effective because bonding and structure data obtained using spectroscopic methods are considered. CD-MUSIC includes multiple sites at which complexation may occur, but unlike the more common Dzombak and Morel (1990) model also takes the spatial distributions of the charges of adsorbed ions into account, i.e., the CD-MUSIC is more chemically based than the Dzombak and Morel (1990) model. The parameters used in the CD-MUSIC model were calibrated as described below.

Adsorption model parameterization to fit the experimental batch equilibrium data was performed using the least square optimization code PEST coupled to the speciation code VMINTEQ v. 3.1 (Gustafsson, 2018). The surface charge parameters were made in the 1-p*K* three-plane model mode. The charge distribution model was used to model the surface acidity and complexation as described by Hiemstra et al. (2007). CD-MUSIC can use the charge distribution to distinguish between inner- and outer-sphere complexation, and between bidentate and monodentate complexes. The net charge introduced in the Stern layer region is distributed using the charge balance $\Delta z_0 = n_0 + n_{H0}$ for the inner Helmholtz layer, and $\Delta z_1 = n_1$ and $\Delta z_2 = n_2$ for the two outer Helmholtz layers, where n is the proportion of the adsorbate ion charge allocated to the respective plane, and n_{H0} is the additional proton eventually introduced to the innermost plane (e.g., for outer-sphere binding). For neutral species like $Si(OH)_4^0$ (aq) and $As(OH)_3^0$ (aq), the sum of the charge distribution values must be zero ($\Delta z_0 + \Delta z_1 + \Delta z_2 = 0$). The same holds for a mono-deprotonated (and therefore negatively charged) oxyanion with one proton added to the surface to give an outer-sphere adsorption complex ($\Delta z_0 + n_{H0} + \Delta z_1 + \Delta z_2 = 0$). One of main benefits of using the complex CD-MUSIC is that the model allows bidentate oxyanion surface coordination to be taken into consideration, as often indicated by spectroscopic analysis results.

The site density for the CD-MUSIC was 6.1 sites nm^{-2} for the $\equiv FeOH^{-0.5}$ hydroxyl groups and 5.3 sites nm^{-2} for the $\equiv Fe_3O^{-0.5}$ hydroxyl groups, as used in previous work (Kersten et al., 2014). The presence of 0.01, 0.05, or 0.1 M $NaNO_3$ as a background electrolyte was taken into consideration through corresponding surface reactions with fixed intrinsic equilibrium reaction constants (Table 1, main text), assuming the formation of symmetrical cation/anion outer-sphere complexes. Capacitance values of C_1 = 0.93 F m^{-2} and C_2 = 0.74 F m^{-2} were fitted using the fixed surface site density and electrolyte ion adsorption constants (Kersten et al., 2014). A point of zero charge of pH_{pzc} = 8.2 and a specific surface area of 300 ± 30 m^2 g^{-1} were used for the GFH. These values were taken from previous publications (Teermann and Jekel, 1999; Kersten et al., 2014). For surface complexes other than complexes with protons and background electrolyte ions, it was assumed that only singly coordinated $FeOH^{-0.5}$ groups were involved. The charge distribution values were not fitted but were fixed at the values suggested for ferrihydrite in the most recent publications cited for the respective oxyanion. This was because the adsorbent phase predominating the surface area in GFH is ferrihydrite, while the other phase (akaganéite) contributed only a minor fraction of the surface area (Kersten et al., 2014).

2.2. Arsenate

The experimental data and model parameterization for arsenate have previously been published (Kersten et al., 2014). The charge distribution coefficients for the two binuclear bidentate surface complexes were the same as suggested for ferrihydrite on the basis of polyhedral geometries optimized by molecular orbital calculations using density functional theory by Hiemstra and Zhao (2016). However, no monodentate surface complex were used because it had insufficient fit quality.

2.3. Phosphate

The technical GFH material is produced using tap water containing Ca, so Ca was leached from the GFH during the batch equilibrium adsorption experiments. The Ca may have interfered with the phosphate adsorption experiments, because sparingly soluble Ca phosphates may have formed. The phosphate adsorption experiments were therefore performed using a separate material charge produced using deionized water, so that the material did not contain Ca. The phosphate concentrations were determined using a Metrohm 920 ion chromatograph. The percentages adsorbed are plotted against the pH for four different phosphate concentrations (0.74–2.0 mmol L^{-1}) equilibrated at three different $NaNO_3$ background electrolyte concentrations (10, 50, and 100 mmol L^{-1}) for 1.0 g L^{-1} GFH (dry weight) in Figure S4. As is typical for oxyanions, the maximum adsorption occurred at low pH values and the amount adsorbed decreased towards neutral and alkaline pH values. Tiberg et al. (2013) found that phosphate adsorption can be modelled using three reactions involving two inner-sphere and one outer-sphere surface complexes:

$$2\equiv FeOH^{-0.5} + 2H^+ + PO_4^{3-} \leftrightarrow (\equiv FeO)_2^{-1+\Delta z_0} PO_2^{\Delta z_1} + 2H_2O \quad (3)$$

$$2\equiv FeOH^{-0.5} + 3H^+ + PO_4^{3-} \leftrightarrow (\equiv FeO)_2^{-1+\Delta z_0} POOH^{\Delta z_1} + 2H_2O \quad (4)$$

$$\equiv FeOH^{-0.5} + 3H^+ + PO_4^{3-} \leftrightarrow \equiv FeOH^{-0.5+\Delta z_0} PO_3^{\Delta z_1} + H_2O \quad (5)$$

The first two surface species are binuclear bidentate surface complexes, one $(\equiv FeO)_2PO_2^{-2}$ deprotonated and the other $(\equiv FeO)_2POOH^-$ protonated. The third is a doubly protonated monodentate surface species ($\equiv FeOHPO_3^{-0.5}$). Hiemstra and Zhao (2016) recently stated that all three surface species are necessary to fit the model to experimental data for a ferrihydrite adsorbent, and they presented charge distribution coefficients derived from polyhedral geometries optimized by performing molecular orbital calculations using density

functional theory. We used the CD values $\Delta z_0 = 0.33$ and $\Delta z_1 = -0.33$ for the monodentate complex calculated by Hiemstra and Zhao (2016), which were somewhat different from the values found by fitting in the earlier study by Tiberg et al. (2013). However, this complex contributed only a minor part in the most acidic pH range, so was not included in our model. Phosphate adsorption can then best be fitted using only the first two surface complexes (Figure S4).

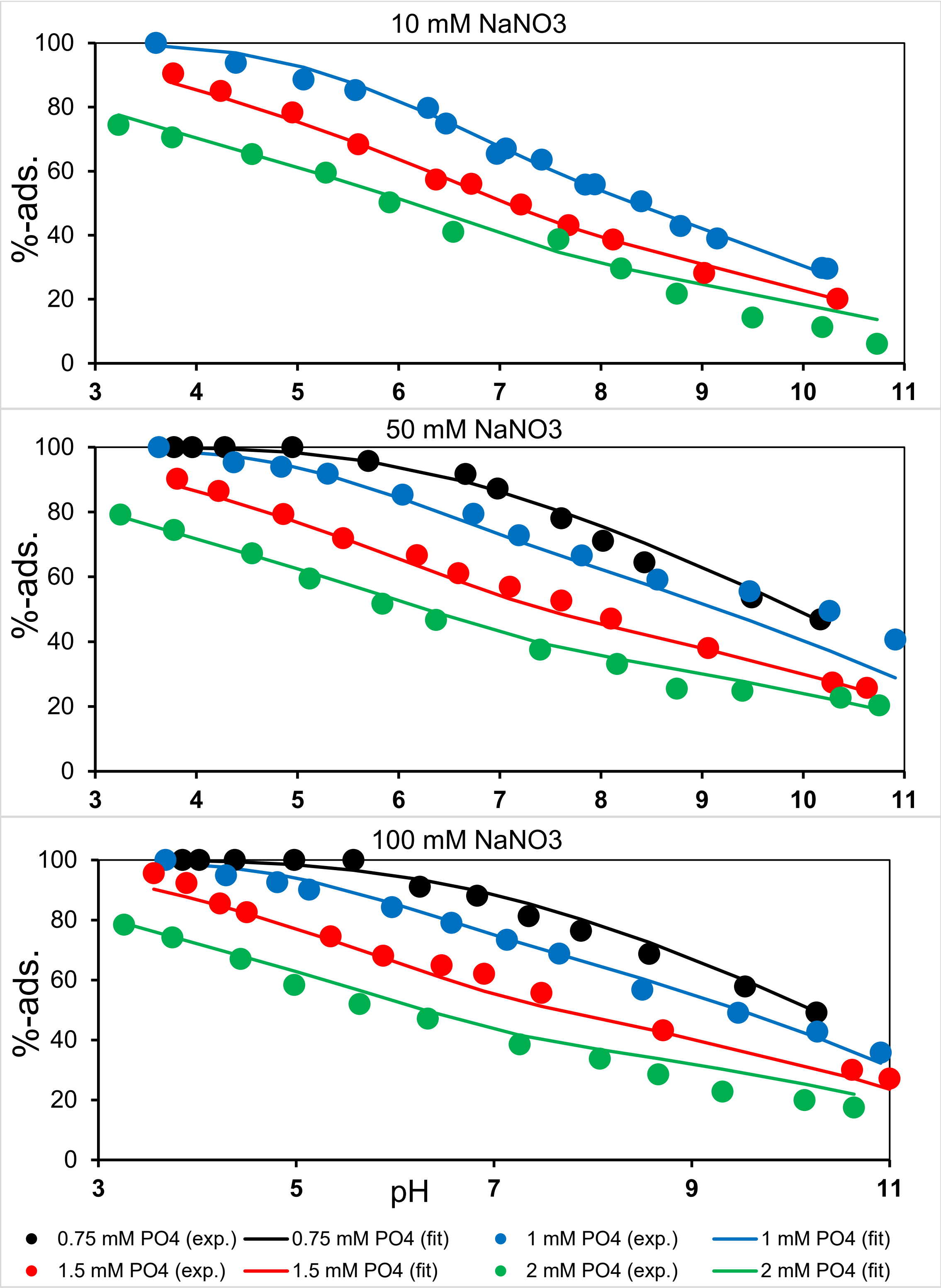

Figure S4. Data and fitted model lines for phosphate adsorption by GFH.

2.4. Silicic acid

The residual silicic acid concentration was determined using the "molybdenum-blue" method. The data for the binary silicic acid–GFH adsorption system shown in Figure S5 were more complicated than data for other oxyanions. As has previously been discussed for the binary silicic acid–goethite system (Kersten and Vlasova, 2009) and silicic acid–ferrihydrite systems (Hiemstra, 2007, 2018), pH-dependent adsorption can be explained through the aqueous speciation of monomeric and polymeric silicic acid species and the hydroxide surface charges. The largest amount of silicic acid adsorbed can be found between the point of zero charge of the GFH (pH_{pzc} = 8.2) and the first dissociation constant of the silicic acid (pK_{a1} = 9.8). Above the first dissociation constant of the silicic acid, there was a steep decrease in the amount adsorbed, suggesting that the role of deprotonated silicic acid in the adsorption reaction was negligible. A less steep but marked decrease below the pH_{pzc} was not caused by a speciation change in the only remaining fully protonated silicic acid species, but by the change in surface charge. The pH dependency was actually related to the amount of charge added to the surface plane, which in turn depended on the structure of the surface complex, as pointed out by Hiemstra et al. (2007). Moreover, strong adsorption of the silicic acid was only slightly affected by the ionic strength. Interestingly, the amount adsorbed decreased slightly as the electrolyte concentration increased the pH < pH_{pzc} range, but increased as the electrolyte concentration increased in the pH > pK_{a1} range. This would have been caused by the increase in the solution ionic strength allowing the charge to be better screened of by the counterions in the electric double layer. Above the pH_{pzc}, more adsorption occurred because of the introduction of a net negative charge and Si adsorption causing less repulsion as the salt concentration increased (Hiemstra et al., 2007). The amount adsorbed therefore decreased below pH_{pzc} because less attraction occurred. The bending in the adsorption curves, particular in the mid-concentration plots shown in Figure S5, therefore did not change, but the maxima of the curves were simply shifted towards slightly higher pH values as the ionic strength increased, as previously found in experiments performed using goethite by Kersten and Vlasova (2009). Only bidentate binuclear inner-sphere adsorption reactions between the singly coordinated surface oxygen groups and the neutral silicic acid molecule were considered:

$$2{\equiv}FeOH^{-0.5} + Si(OH)_4^0 \leftrightarrow {\equiv}(FeO)_2^{-1+\Delta z_0}\,Si(OH)_2^{\Delta z_1} + 2H_2O \qquad (6)$$

This reaction was previously suggested for ferrihydrite (Hiemstra, 2018), being the main surface-contributing compound in GFH. Hiemstra et al. (2007) and Hiemstra (2018) found that silicic acid tends to polymerize on surfaces at high Si concentrations (>1 mmol L^{-1}). This was probably because 10-times higher total Si concentrations were used than in our previous work using goethite. A maximum surface loading of 3.0 μmol m^{-2} was found in this study. This was below the theoretical maximum calculated from the site density of singly coordinated oxygen surface groups on the GFH (5.0 μmol m^{-2}). Nonetheless, adsorption reactions for two oligomer species (trimer, n = 3, and tetramer, n = 4) were therefore used in addition to include this effect (Hiemstra et al., 2007; Hiemtra, 2018), with one proton removed from one of the free OH ligands of the adsorbed silicic acid oligomer:

$$2{\equiv}FeOH^{-0.5} + nSi(OH)_4^0 \leftrightarrow {\equiv}(FeO)_2^{-1+\Delta z_0}\,Si_nO_{n-1}(OH)_{2n-1}O + H_2O \qquad (7)$$

The charge distribution was fixed, as suggested by Hiemstra (2018) from density functional theory models. Fitting with the code VMINTEQ-PEST (Gustafsson, 2018) gave a unique set of three surface complexation constants with an uncertainty of ±0.1 log units. This best reproduced the 3D data-set for the pH, ionic strength, and Si concentration variations with quite a good match (R = 0.99; solid lines in Figure S5). The log$K$ values calculated this way were a bit lower than the values reported by Hiemstra (2018) for goethite, but appeared reasonable given that the GFH pH_{PZC} by one unit lower than the goethite pH_{PZC}.

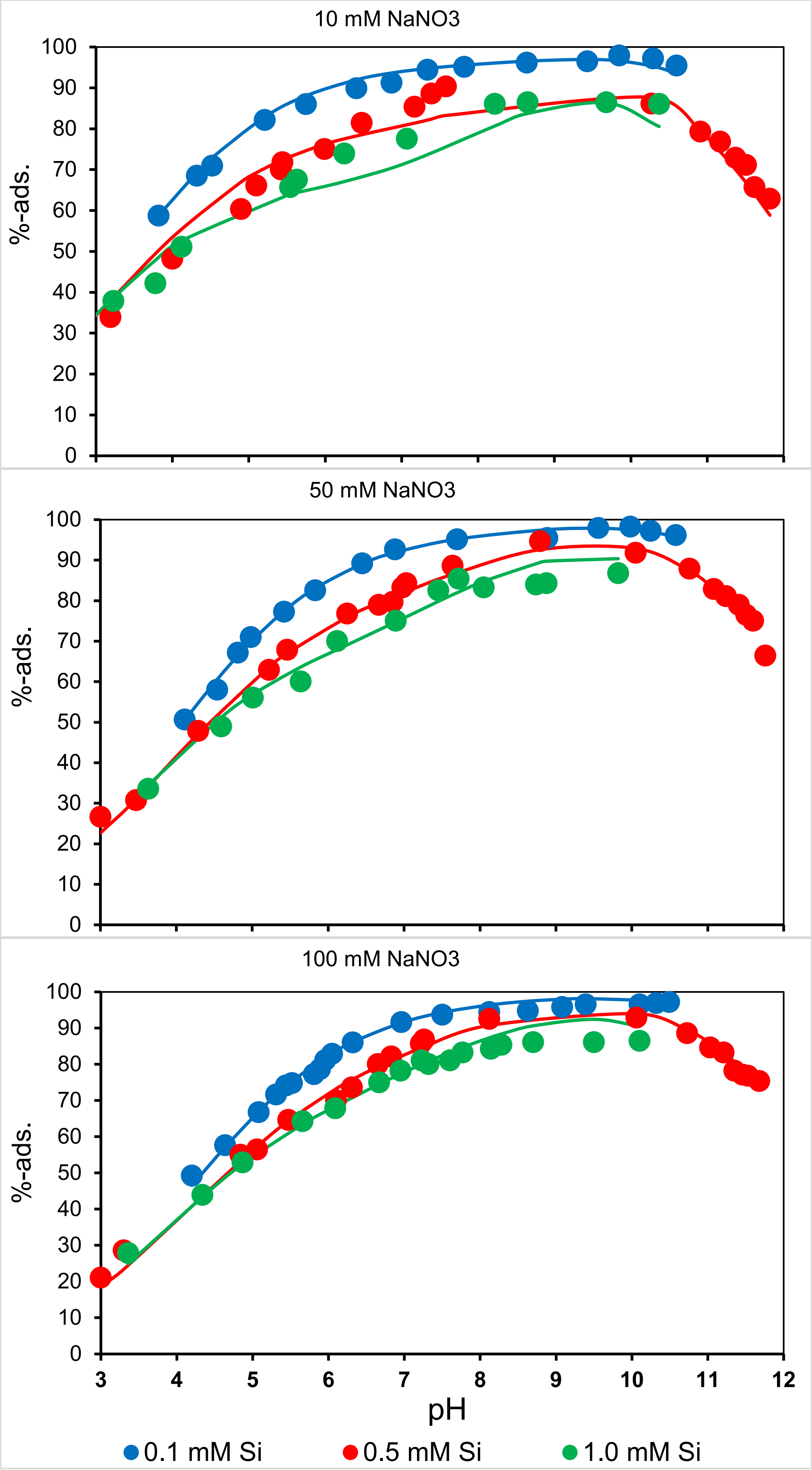

Figure S5. Silicic acid adsorption plotted against pH for GFH. The dots are the experimental data and the solid lines are the data fitted using the CD-MUSIC model.

The species distributions are compared in Figure S6. The monomer was dominant at lower Si concentrations, but oligomers were dominant at higher Si concentrations, as was found in the distributions shown in Figure 10 in the publication by Hiemstra (2018).

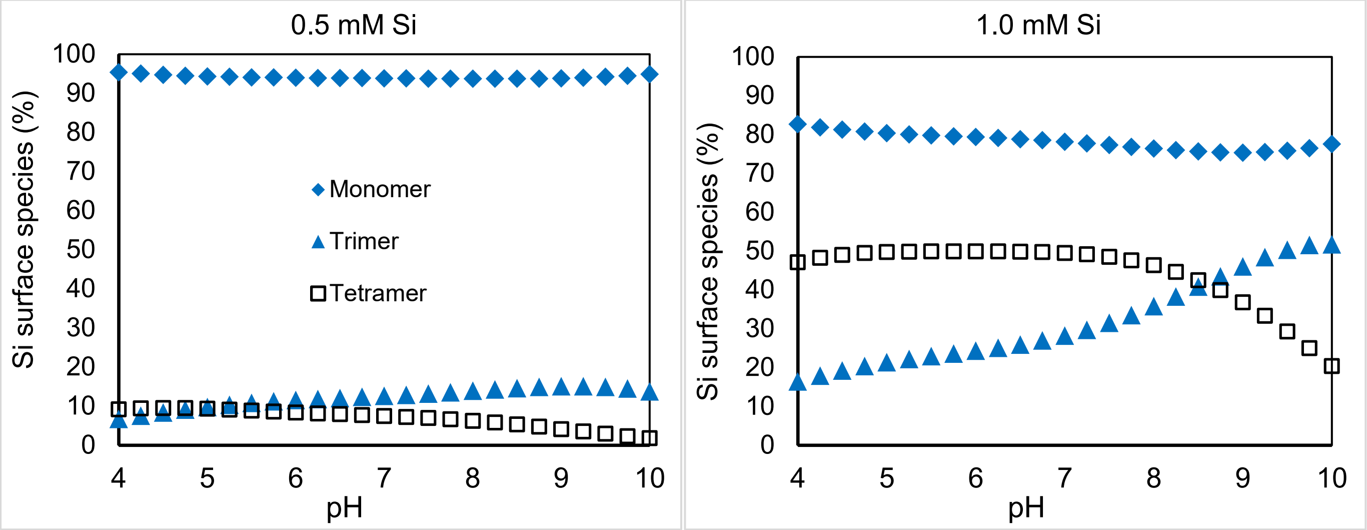

Figure S6. pH-dependence of the surface species distributions of Si adsorbed to GFH at a low (0.5 mM) and a high (1.0 mM) total concentration at equilibrium in 0.1 M $NaNO_3$, calculated using our CD-MUSIC model parameter.

3. Published data re-fitted using the CD-MUSIC model

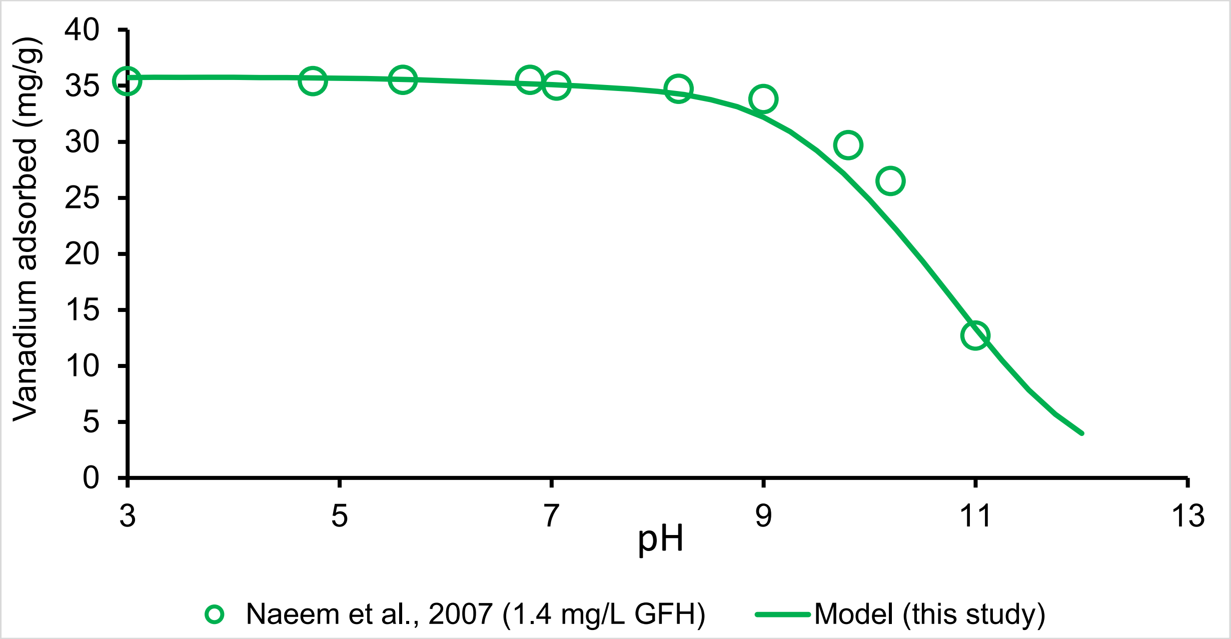

Figure S7. Experimental results (green dots) for 50 mg L^{-1} vanadate adsorption onto 1.4 g L^{-1} GFH, previously published by Naeem et al. (2007). The green curve is not fitted, but is an excellent model prediction achieved using the CD-MUSIC parameters from this study. Naeem et al. (2007) published also experimental data for a lower GFH concentration (0.35 g L^{-1}). However, the data were somewhat erroneous, because a maximum V load of 160 g L^{-1} could not be reached with an initial V concentration of 50 mg L^{-1} and a maximum adsorption capacity Q_m of 111 mg g^{-1}.

Now verified, the surface complexation model allows competition between oxyanions already reported in literature to be predicted. This is shown in Figure S8 using experimental data published by Kolbe et al. (2011). In these experiments performed at pH 7.5, arsenate was outcompeted more by vanadate than by other oxyanions such as antimonite and phosphate. The data were predicted well using our CD-MUSIC surface complexation model (solid curves in Figure S8).

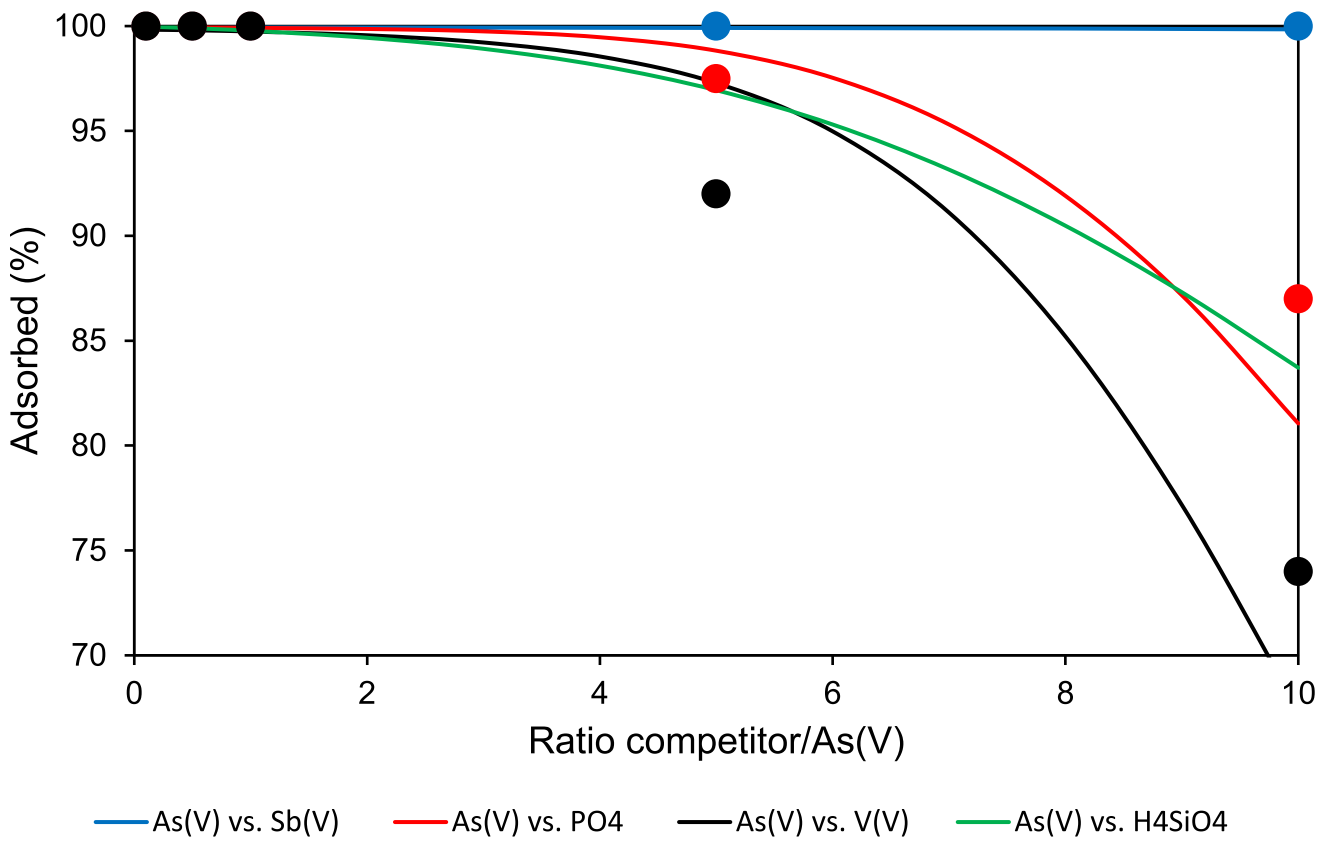

Figure S8. Influences of competitive adsorption of oxyanions on arsenate adsorption to GFH at pH 7.5. The dots show experimental data published by Kolbe et al. (2011) for As(V) vs. Sb(V), As(V) vs. PO_4^{-3}, and As(V) vs. V(V). The lines were calculated using our CD-MUSIC model. The first three black dots near the y-axis cover blue and red dots, which therefore cannot be seen.

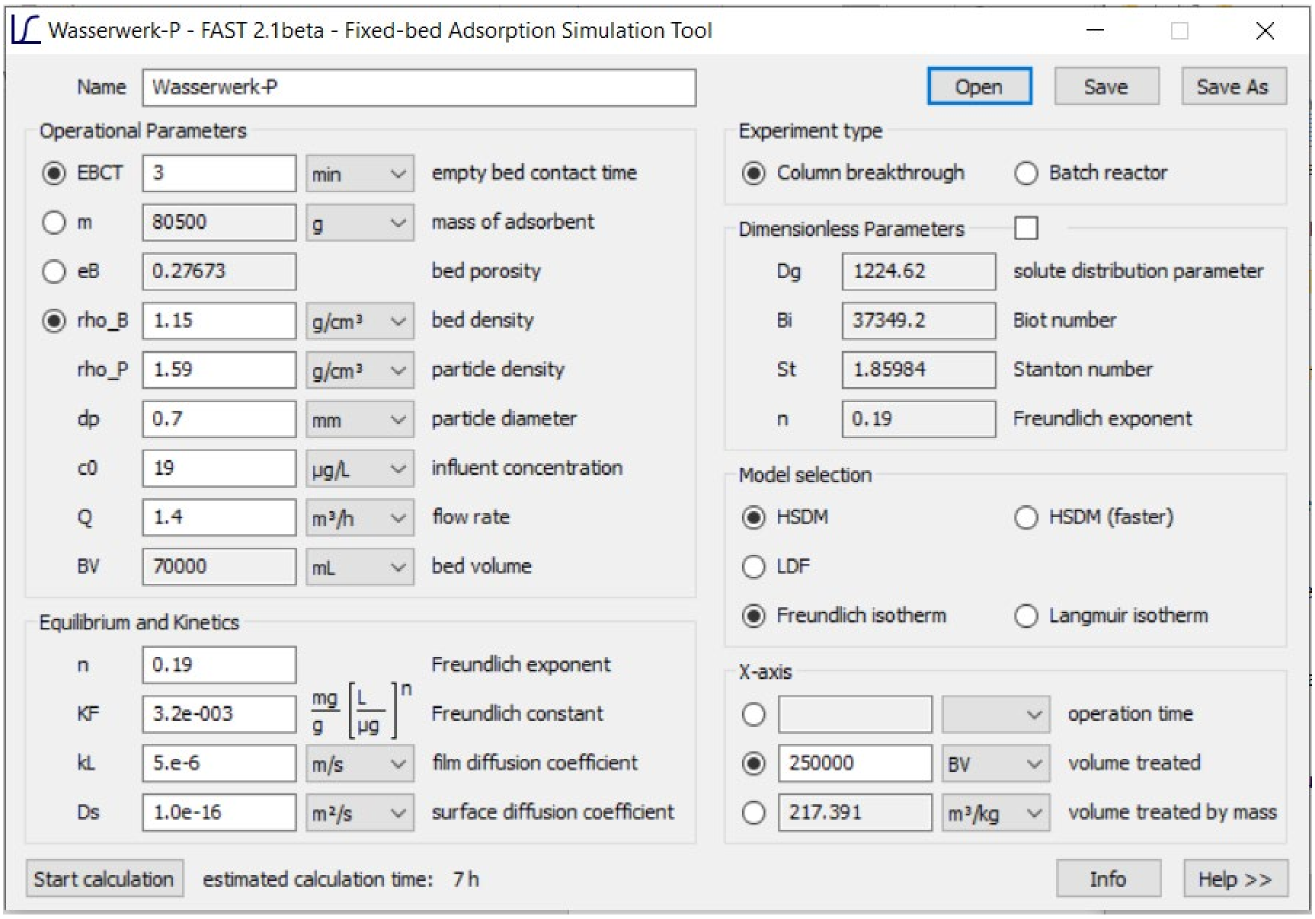

Figure S9. Screenshot of the FAST v.2.1 software code used for BTC simulations.

References

Dzombak, D.A., Morel, F.M.M., (1990). Surface Complexation Modeling: Hydrous Ferric Oxide. John Wiley & Sons, New York.

Gustafsson, J.P., 2018. Visual MINTEQ 3.1, available at https://vminteq.lwr.kth.se

Gustafsson, J.P.: Vanadium geochemistry in the biogeosphere – speciation, solid-solution interactions, and ecotoxicity. Applied Geochemistry **102**, 1-25 (2019)

Hiemstra T., Van Riemsdijk W.H., 2006. On the relationship between charge distribution, surface hydration, and the structure of the interface of metal hydroxides" Colloid Interface Sci. 301, 1-18.

Hiemstra, T., Barnett, M.O., van Riemsdijk, W.H., 2007. Interaction of silicic acid with goethite. J. Colloid Interf. Sci. 310, 8-17.

Hiemstra, T., Zhao, W., 2016. Reactivity of ferrihydrite and ferritin in relation to surface structure, size, and nanoparticle formation studied for phosphate and arsenate. Environ. Sci.: Nano 3, 1265-1279.

Hiemstra, T., 2018. Ferrihydrite interaction with silicate and competing oxyanions: Geometry and hydrogen bonding of surface species. Geochim. Cosmochim. Acta 238, 453-476.

Kersten, M., Vlasova, N., 2009. Silicate adsorption by goethite at elevated temperatures. Chem. Geol. 262, 372-379.

Kersten, M., Karabacheva, S., Vlasova, N., Branscheid, R., Schurk, K., Stanjek, H., 2014. Surface complexation modeling of arsenate adsorption by akaganéite (β-FeOOH)-dominant granular ferric hydroxide. Colloids and Surfaces A 448, 73-80.

Kinniburgh, D.G., Cooper, D.M. PhreePlot – Creating graphical output with Phreeqc, 598 S. (updated 2018: http://www.phreeplot.org)

Kolbe, F., Weiss, H., Morgenstern, P., Wennrich, R., Lorenz, W., Schurk, K., Stanjek, H., Daus, B., 2011. Sorption of aqueous antimony and arsenic species onto akaganéite. Journal of Colloid and Interface Science 357, 460-465.

Li, X., Reich, T., Kersten, M., Jing, C., 2019. Low-molecular-weight organic acid complexation affects antimony(III) adsorption by granular ferric hydroxide. Environ. Sci. Technol. 53, 5221-5229.

Naeem, A., Westerhoff, P., Mustafa, S., 2007. Vanadium removal by metal (hydr)oxide adsorbents. Water Res. 41, 1596-1602.

Teermann, I.P., Jekel, M.R., 1999. Adsorption of humic substances onto β-FeOOH and its chemical regeneration. Wat. Sci. Tech. 40, 199-206.

Tiberg, C., Sjöstedt, C., Persson, I., Gustafsson, J.P., 2013. Phosphate effects on copper(II) and lead(II) sorption to ferrihydrite. Geochim. Cosmochim. Acta 120, 140-157.
